# Clinical evaluation of stereotactic radiation therapy for recurrent or second primary mediastinal lymph node metastases originating from non-small cell lung cancer

**DOI:** 10.18632/oncotarget.3704

**Published:** 2015-03-30

**Authors:** Mao-Bin Meng, Huan-Huan Wang, Nicholas G. Zaorsky, Xian-Zhi Zhao, Zhi-Qiang Wu, Bo Jiang, Yong-Chun Song, Hong-Qing Zhuang, Feng-Tong Li, Lu-Jun Zhao, Chang-Li Wang, Kai Li, Ping Wang, Zhi-Yong Yuan

**Affiliations:** ^1^ Department of Radiation Oncology, CyberKnife Center, and Key Laboratory of Cancer Prevention and Therapy, Tianjin Medical University Cancer Institute and Hospital, National Clinical Research Center for Cancer, Tianjin, China; ^2^ Department of Radiation Oncology, Fox Chase Cancer Center, Philadelphia, PA, USA; ^3^ Department of Lung Cancer and Key Laboratory of Cancer Prevention and Therapy, Tianjin Medical University Cancer Institute and Hospital, National Clinical Research Center for Cancer, Tianjin, China; ^4^ Department of Thoracic Oncology and Key Laboratory of Cancer Prevention and Therapy, Tianjin Medical University Cancer Institute and Hospital, National Clinical Research Center for Cancer, Tianjin, China

**Keywords:** local control, mediastinum, non-small cell lung cancer, stereotactic body radiation therapy, fractionated stereotactic radiation therapy

## Abstract

**Aims:**

To evaluate the safety and efficacy of stereotactic radiotherapy (SRT, both stereotactic body RT [SBRT] and fractionated stereotactic RT [FSRT]) in the treatment of patients with recurrent or second primary mediastinal lymph node metastases (R/SP-MLNMs) originating from non-small cell lung cancer (NSCLC).

**Methods:**

Between 10/2006 and 7/2013, patients with R/SP-MLNMsoriginating from NSCLC were enrolled and treated with SRT at our hospital; their data was stored in prospectively-collected database. The enrolled patients were divided into Group A (without prior RT) and Group B (with prior RT). The primary end-point was overall survival (OS). The secondary end-points were the MLNM local control (LC), the time to symptom alleviation, and toxicity using the Common Terminology Criteria for Adverse Events (CTCAE v4.0).

**Results:**

Thirty-three patients were treated (16 in Group A with 19 R/SP-MLNMs and 17 in Group B with 17 R/SP-MLNMs). For the entire cohort, the median OS was 25.5 months with a median follow-up of 20.9 months (range, 3.2-82). The 1-year and 3-year actuarial LC rates were 100% and 86%, respectively. Symptom alleviation was observed in 52% of patients, after a median of 6 days (range, 3-18). CTCAE v4.0 ≥ Grade 3 toxicities occurred in 5 patients (15%; all in Group B); among them, Grade 5 in 2 patients.

**Conclusions:**

We recommend exercising extreme caution in using SRT for R/SP-MLNMs in patients who received prior RT (particularly to LN station 7). For patients without previous RT, SRT appears to be safe and efficacious treatment modality; prospective studies are warranted.

## INTRODUCTION

Patients treated with definitive surgical resection for early-stage non-small cell lung cancer (NSCLC) have a 25-35% rate of nodal metastasis at the time of surgery [1-2]. Similarly, among patients receiving conventionally fractionated external beam radiation therapy (EBRT; with 3D conformal RT [3D-CRT], or intensity modulated RT [IMRT]), the regional nodal failure rate, even when these nodes are not intentional targets, is 5-15% [3-7]. Recurrent or second primary mediastinal lymph node metastases (R/SP-MLNMs) are challenging for physicians to treat, given their proximity to critical structures (e.g. esophagus, great vessels, and trachea) [8]. Moreover, R/SP-MLNMs (unlike primary parenchymal tumors) are more likely to negatively impact patient quality of life by causing symptoms (e.g. dysphagia, dyspnea, pain) [9].

Currently, there is no standard approach for managing R/SP-MLNMs. Generally, surgical salvage of R/SP-MLNMs is not always feasible because of disease extent or the involvement of critical structures [10]. The National Comprehensive Cancer Network (NCCN) recommends chemo-RT for patients with R/SP-MLNMs if the patients did not receive RT (RT may be delivered as palliative 3D-CRT); and chemo alone if the patients received previous RT. Notably, there is a lack of level I evidence in most situations [11-13]. Palliative EBRT is typically delivered in 2-3 Gy fractions to a total of 30 Gy; unfortunately, the dose is limited because of a relatively high dose of radiation deposited to surrounding structures, causing toxicity.

**Figure 1:**
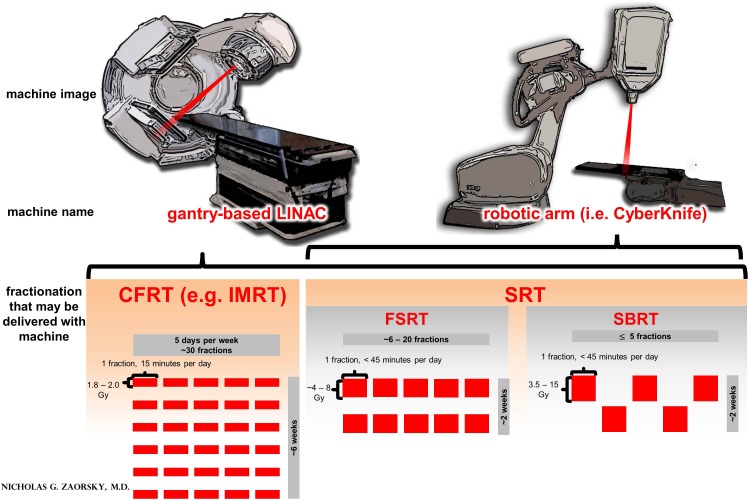
A comparison of treatment machines and fractionation options in external beam radiation therapy (EBRT) for non-small cell lung cancer (NSCLC) Legend: typically, in the primary treatment of NSCLC, EBRT is delivered as conventionally fractionated RT (CFRT), which is 1.8 – 2.0 Gy per day, one fraction per day, for a total of ~30 fractions, to a total dose of ~60 Gy; CFRT is delivered with intensity modulated RT (IMRT). Stereotactic radiation therapy (SRT) is a type of EBRT that delivers RT accurately and precisely to the tumor, more so than CFRT with IMRT. SRT may be used for small (i.e. T1-2) or recurrent / second primary mediastinal lymph node metastases (R/SP-MLNMs, as in the current work). SRT is divided into stereotactic body RT (SBRT, the delivery of 3.5-15 Gy per fraction, in 5 fractions or less) and fractionated stereotactic RT (FSRT, in more than 5 fractions). SRT may be delivered with a gantry-based LINAC or with a robotic arm LINAC (i.e. a CyberKnife).

Stereotactic radiation therapy (SRT) is a type of EBRT that delivers RT accurately and precisely to the tumor, more so than conventionally fractionated IMRT. SRT is divided into stereotactic body RT (SBRT, the delivery of 3.5-15 Gy per fraction, in 5 fractions or less) and fractionated stereotactic RT (FSRT, in more than 5 fractions). The fractionation schemes and treatment machines are juxtaposed in Figure 1. SRT can be delivered either using a traditional linear accelerator or using a robotic arm (i.e. CyberKnife). SRT appears to be an acceptable treatment option for recurrent tumors, and several series report high rates of local control and low incidence of complication in SRT for re-RT of NSCLC [14-18]. We hypothesized that SRT (both FSRT and SBRT) was a safe and efficacious treatment modality for R/SP-MLNMs from NSCLC.

**Figure 2:**
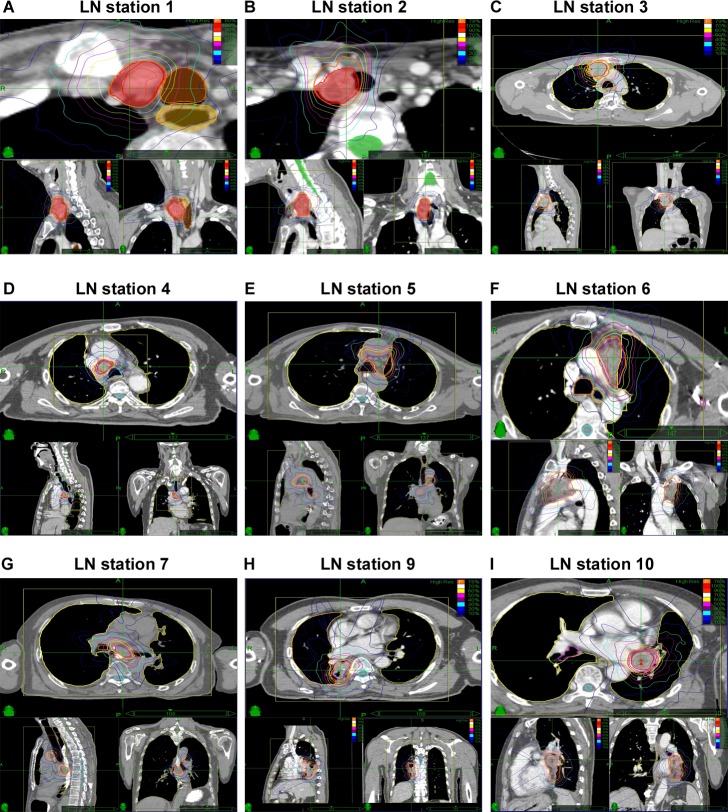
Representative planning CT and isodose distributions with SRT of patients with R/SP-MLNMs originating from NSCLC The purple and yellow lines indicate GTV and PTV, respectively. (**A**-**I**) The LN stations of MLNMs. CT: computer tomography; SRT: stereotactic radiotherapy; R/SP-MLNM: recurrent / second primary mediastinal lymph node metastases; GTV: gross tumor volume; PTV: planning target volume.

**Figure 3:**
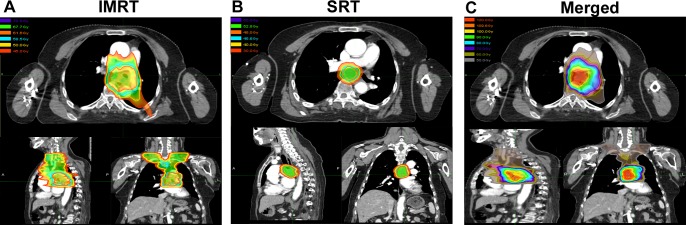
Analysis of SRT, IMRT, and the composite images for a representative patient In this case, a 64-year-old woman squamous cell lung cancer located in left lower lobe with station 7 MLN, received SRT 6.8 months after completion of IMRT;. Unfortunately, the patient died of tracheoesophageal fistula six weeks after completion of SRT. (**A**) IMRT was delivered in in 28 fractions to a dose of 61.6 Gray for NSCLC of LN station 7; (**B**) At 6.8 months after completion of IMRT, there was a R-MLNM at station 7; SRT was initiated, in 8 fractions to 48 Gy, prescribed to the 75% isodose line; (**C**) Composite plans were created of SRT and IMRT using MIM Software. SRT: stereotactic radiotherapy; IMRT: intensity modulated radiation therapy.

## RESULTS

### Patient characteristics

Clinical information on 1,765 patients treated with SRT between October 1 2006 and January 13 2013 at the CyberKnife Center of Tianjin Medical University Cancer Institute & Hospital was reviewed. Of these patients, there were 1421 who had NSCLC and were initially evaluated by a multidisciplinary tumor board at our center. There were 33 patients with 36 R/SP-MLNMs from NSCLC were subsequently treated with SRT. The patients were divided into Group A (without prior RT; 16 patients with 19 R/SP-MLNMs) and Group B (with prior RT, 17 patients with 17 R/SP-MLNMs). Sixteen patients had 18 R-MLNMs, and 17 patients had 18 SP-MLNMs. For group B, the median interval between initial RT and re-RT was 6.7 months (range, 1.1-39.4 months). Table 1 lists patient characteristics. Table 2 lists the MLN station involvement of the patients in this study. Table 3 lists the RT doses, EQD2s, and PTVs of group B.

**Table 1:** Summary of patient characteristics

Parameter	Group A (16 pts)	Group B (17 pts)	All (33 pts)
Age (years)			
< 60	7 (44%)	8 (47%)	15 (45%)
≥ 60	9 (57%)	9 (53%)	18 (55%)
Gender			
Male	11 (69%)	12 (71%)	23 (70%)
Female	5 (31%)	5 (29%)	10 (30%)
Pathology of primary cancer			
Squamous cell carcinoma	10 (63%)	7 (41%)	17 (52%)
Adenocarcinoma	6 (37%)	4 (24%)	10 (30%)
Large cell carcinoma	0	2 (12%)	2 (6%)
Other^†^	0	4 (23%)	4 (12%)
Location of primary cancer			
Right upper lobe	6 (38%)	2 (12%)	8 (24%)
Right middle lobe	1 (6%)	1 (6%)	2 (6%)
Right lower lobe	2 (13%)	3 (18%)	5 (15%)
Left upper lobe	5 (31%)	7 (41%)	12 (36%)
Left lower lobe	2 (12%)	4 (23%)	6 (19%)
Radiographic appearance of primary cancer			
Nodular	13 (81%)	16 (94%)	29 (85%)
Infiltrative	3 (19%)	1 (6%)	4 (15%)
Initial stage^‡^			
I/II	10 (63%)	1 (6%)	11 (33%)
IIIA	6 (37%)	5 (29%)	11 (33%)
IIIB	0	11 (65%)	11 (34%)
No. of R/SP-MLNMs			
1	14 (88%)	16 (94%)	30 (91%)
>1	2 (12%)	1 (6%)	3 (9%)
MLNM type			
Recurrence	5 (31%)	12 (71%)	17 (52%)
Second primary	11 (69%)	5 (29%)	16 (48%)
Clinical symptoms of R/SP-MLNMs			
Yes	5 (31%)	12 (71%)	17 (52%)
No	11 (69%)	5 (29%)	16 (48%)
Synchronous metastases			
Yes	5 (31%)	3 (18%)	8 (24%)
No	11 (69%)	14 (82%)	25 (76%)
Radiographic diagnosis R/SP-MLNMs			
PET-CT	9 (56%)	10 (59%)	19 (58%)
CT	7 (44%)	7 (41%)	14 (42%)
Recurrent staging^‡^			
II	1 (6%)	0	1 (3%)
III	10 (63%)	14 (82%)	24 (73%)
IV	5 (31%)	3 (18%)	8 (24%)
Time to recurrence (months)			
< 15.5	7 (44%)	4 (24%)	11 (33%)
≥ 15.5	9 (56%)	13 (76%)	22 (67%)
Therapy prior to SRT^§^			
S	16 (100%)	11 (65%)	27 (82%)
CT	10 (63%)	14 (82%)	24 (73%)
RT	0	17 (100%)	17 (52%)
MTT	1 (6%)	0	1 (3%)
Therapy after SRT^§^			
CT	6 (38%)	8 (47%)	14 (42%)
MTT	1 (6%)	2 (12%)	3 (9%)
None	10 (63%)	7 (41%)	17 (52%)

Note: Group A: Patients with R/SP-MLNMs who received treatment including surgery and/or chemotherapy, but not radiation therapy; Group B: Patients with R/SP-MLNMs who received treatment including surgery and/or chemotherapy, and radiation therapy

† Other is defined as the various combinations of squamous cell carcinoma, adenocarcinoma, and large cell carcinoma.

‡ The AJCC (6th edition) was used for staging.

§ Some patients had more than one therapy.

Abbreviations: Pts: patients; R/SP-MLNMs: recurrent or second primary mediastinal lymph node metastases; PET-CT: positron emission tomography/computed tomography; CT: computed tomography; SRT: stereotactic radiation therapy; S: surgery; CT: chemotherapy; RT: radiotherapy; MTT: molecular targeted therapy.

**Table 2:** LN stations of R/SP-MLNMs

Nodal zone	Group A (16 pts)^†^	Group B (17 pts)^‡^	All (33 pts)
Upper	9 (47%)	7 (41%)	16 (44%)
1R	1	0	1
1L	0	1	1
2R	1	0	1
2L	0	1	1
3A	1	0	1
3P	1	0	1
4R	4	2	6
4L	1	3	4
Aorticopulmonary	3 (16%)	4 (24%)	7 (19%)
5	1	2	3
6	2	2	4
Subcarinal	2 (11%)	4 (24%)	6 (17%)
7	2	4	6
Lower	1 (5%)	1 (6%)	2 (6%)
9R	1	1	2
9L	0	0	0
Hilar-interlobar	4 (21%)	1 (5%)	5 (14%)
10R	2	0	2
10L	2	1	3
All	19 (100%)	17 (100%)	36 (100%)

Note: Group A: Patients with R/SP-MLNMs who received treatment including surgery and/or chemotherapy, but not radiation therapy; Group B: Patients with R/SP-MLNMs who received treatment including surgery and/or chemotherapy, and radiation therapy

† There were three patients with more than one R/SP-MLNM. Among these patients, one patient with R/SP-MLNMs had involvement of the 4R and 9 stations, one patient with R/SP-MLNMs had involvement of the 3A and 6 stations, and one patient with R/SP-MLNMs had involvement of the 1R, 2R, 3P, and 7 stations, respectively.

‡ There was one patient with R/SP-MLNMs with involvement of the stations 5 and 7.

Abbreviations: R/SP-MLNMs: recurrent/second primary mediastinal lymph node metastases; Pts: patients; R: right; L: left.

**Table 3:** An overview of the treatment schedules for NSCLC patients with R/SP-MLNMs receiving SRT (Group B)

	Re-irradiation					Interval	First radiation					Total dose (sum of previous doses)	
Patient initials	Total dose (Gy)	No. of fractions	EQD2 α/β = 10	EQD2 α/β = 3	PTV (mL)	months	Total dose (Gy)	No. of fractions	EQD2 α/β = 10	EQD2 α/β = 3	PTV (mL)	EQD2 α/β = 10	EQD2 α/β = 3
WYC	30	6	45	80	26.19	4.1	42	21	50.4	70	-	95.4	150
WJF	60	30	72	100	-	3.1	50	10	75	133	28.30	147	233
TBW	60	5	132	300	17.89	6.3	60	30	72	100	-	204	400
LSS	1.8	1	2.12	2.88	-	9.0	45	10	65.25	112.5	15.89	67.37	115.35
TYJ	60	15	84	140	7.95	9.1	50	20	62.5	91.67	-	146.5	231.67
ZXM	24	4	38.4	72	15.85	5.5	60	30	72	100	-	110.4	172
ZXL	36	4	68.4	144	50.63	1.1	29.2	7	41.38	69.79	-	109.78	213.79
ZXZ	40	7	62.86	116.19	144.93	5.9	63	30	76.23	107.1	-	139.09	223.29
LJX	30	10	39	60	71.84	6.5	45	18	56.25	82.5	-	95.25	142.5
LYS	42	6	71.4	140	12.90	7.3	50	25	60	83.33	-	131.4	223.33
DXR	61.6	28	75.15	106.77	-	19.1	48	8	76.8	144	32.52	151.95	250.77
DJG	67.5	27	84.38	123.75	-	39.4	45	5	85.5	180	50.22	169.88	303.75
WFL	60	30	72	100	-	6.7	40	5	72	146.67	3.70	144	246.67
WSM	45	6	78.75	157.5	20.02	8.1	60	30	72	100	-	150.75	257.5
CGL	45	5	85.5	180	15.72	7.8	64	32	76.8	106.67	-	162.3	286.67
ZGL	54	3	151.2	378	11.90	6.3	60	30	72	100	-	223.2	478
LEP	36	3	79.2	180	30.27	17.8	66	33	79.2	110	-	158.4	290

Abbreviations: EQD2: Equivalent dose in 2 Gray per fraction, based on the formuland ((d+α/β)/(2+α/β)), where n is number of fractions, and d is dose/fraction (Gy); GTV: Gross tumor volume; PTV: Planning target volume.

### Treatment characteristics

**Figure 4:**
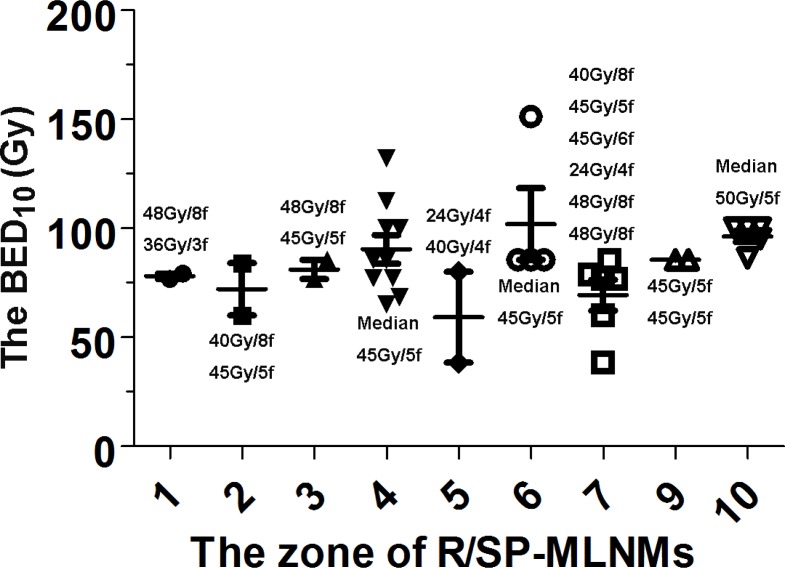
The detailed summary of the prescribed dose, dose per fraction, and BED_10_ from each MLNM stations BED_10_: biologically effective dose at an α/β of 10; R/SP-MLNMs: recurrent or second primary mediastinal lymph node metastases; Gy: Gray; f: fraction.

The detailed summary of the treatment planning parameters for all patients and each MLNM station are listed in Table 4 and Figure 4. For the whole cohort, the median PTV was 17.89 mL (range, 4.0–145.0 mL). Patients received a median of 5 fractions (range, 3 to 15 fractions) with a median dose of 8 Gy per fraction (range, 3-18 Gy), and a total dose of 45 Gy (range, 24-60 Gy). The median BED was 83.30 Gy (range, 38–151 Gy). The dose was prescribed to the median 77% isodose line (range, 70–83%), which encompassed 95% of the PTV.

**Table 4:** Summary of SRT treatment parameters

	Group A (16 pts)	Group B (17 pts)	All (33 pts)
Parameter (unit)	median (range)	median (range)	median (range)
PTV (mL)	11.81 (7-86)	23.11 (4-145)	17.89 (4-145)
Prescription dose (Gy)	48 (35-56)	41 (24-60)	45 (24-60)
Number of fractions	5 (3-8)	6 (3-15)	5 (3-15)
Dose per fraction (Gy)	9 (6-15)	6 (3-18)	8 (3-18)
BED_10_ (Gy)	85.50 (60-112)	71.70 (38-151)	83.30 (38-151)
Prescription isodose line, %	77 (70-83)	76 (72-81)	77 (70-83)

Note: Group A: Patients with R/SP-MLNMs who received treatment including surgery and/or chemotherapy, but not radiation therapy; Group B: Patients with R/SP-MLNMs who received treatment including surgery and/or chemotherapy, and radiation therapy

Abbreviations: SRT: stereotactic radiation therapy; Pts: patients; PTV: planning target volume; Gy: Gray; BED_10_: biologically effective dose at α/β value of 10.

### Overall survival

**Figure 5:**
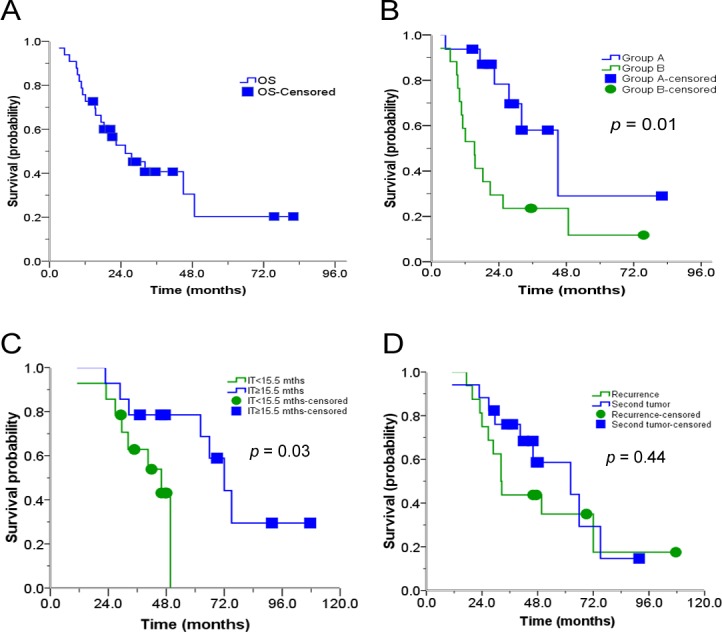
Actuarial OS of patients (**A**) OS after receiving SRT; (**B**) OS after receiving SRT depending on treatment group (Group A is without prior RT; Group B is with prior RT); (**C**) OS after receiving SRT, depending on the time between surgery and SRT; (**D**) OS after receiving SRT, depending on R- vs. SP-MLNMs. OS: Overall survival; R/SP-MLNM: recurrent /second primary mediastinal lymph node metastases; SRT: stereotactic radiation therapy; S: surgery; IT: interval time.

For the whole cohort, the median follow-up was 20.9 months (range, 3.2-82). The median OS was 25.5 months; with the 1-year, 3-year, and 5-year OS rates of 72.7%, 40.7%, and 20.4%, respectively (Figure 5A). Compared to patients in Group B, patients in Group A had significantly longer median OS (15.3 months vs. 45.0 months, *p* = 0.01, Figure 5B). For patients who received SRT < 15.5 months after their surgery, the median OS was 42.0 months vs. 72.0 months for those treated at a ≥ 15.5 months interval (*p* = 0.03, Figure 5C). Patients who presented with a R-MLNMs had a median OS of 32.2 months, with 3-year survival rate of 43.8% vs. 62.2 months and 68.4% for patients with SP-MLNMs (*p* = 0.44, Figure 5D). In addition, the OS showed a slight trend towards superiority of SRT with chemo over SRT without chemo, although these differences were not statistically significant (*p* = 0.35).

### R/SP-MLNM response

Twenty one patients (21/33, 64%) had a CR, 11 patients (11/33, 33%) had a PR, and 1 patient (1/33, 3%) had no response. The 1-year and 3-year actuarial LC rates for all eligible patients were 100% and 86%, respectively. The rates of CR and locoregional control were better in patients with SP-MLNMs vs. those with R-MLNMs (*p* = 0.02).

### The time to symptom alleviation

The most common symptoms were cough, shortness of breath, hoarseness, and difficulty swallowing. An improvement in symptoms was observed after a median follow-up of 6 days (range, 3-18) in 52% of patients. Symptom alleviation remained throughout the follow-up period.

### Patterns of failure

No patient failed within the R/SP-MLNM PTV. Five patients (5/33, 15%) had no progression after SRT (all in Group A); there were 17 patients (17/33, 52%; 5 in Group A and 12 in Group B) who had progression, with a median of 16.9 months after SRT (range, 2.7-75.5 months). Among the patients with progression, one patient (with station 7 R-MLNM in Group B) had diffuse progression including regional failure. The remaining patients had distant metastases to liver, lung, bone, brain, and non-regional lymph nodes.

### Toxicities

Toxicity of all patients is summarized in Table 5. Six patients (18%) experienced CTCAE v4.0 Grade 1 to 2 acute toxicities including pneumonitis, esophagitis, tracheitis, chest pain, agranulocytosis, and thrombocytopenia. Three patients (9%) experienced Grade 3 acute toxicities including esophagitis and tracheitis. Almost all of these acute toxicities occurred in Group B, and they were generally transient and resolved with conservative management. Late radiation toxicities were observed in 4 patients (12%), all in Group B; 2 patients (6%) died from Grade 5 late toxicities. Both patients were treated to LN station 7. One patient died of tracheoesophageal fistula five weeks after completion of re-RT, and the second patient died of tracheoesophageal fistula six weeks after completion of re-RT.

**Table 5:** Toxicities of patients with R/SP-MLNMs treated with SRT

	Group A, n (%)				Group B, n (%)				Total, n (%)			
Acute toxicities	Any Grade	Grade 3	Grade 4	Grade 5	Any Grade	Grade 3	Grade 4	Grade 5	Any Grade	Grade 3	Grade 4	Grade 5
Pneumonitis	0	0	0	0	1 (6)	0	0	0	1 (3)	0	0	0
Esophagitis	1 (6)	0	0	0	1 (6)	1 (6)	0	0	3 (9)	1 (3)	0	0
Tracheitis	0	0	0	0	2 (12)	2 (12)	0	0	2 (6)	2 (6)	0	0
Chest pain	0	0	0	0	1 (6)	0	0	0	1 (3)	0	0	0
Agranulocytosis	0	0	0	0	1 (6)	0	0	0	1 (3)	0	0	0
Thrombocytopenia	0	0	0	0	1 (6)	0	0	0	1 (3)	0	0	0
**Late toxicities**												
Tachycardia	0	0	0	0	1 (6)	0	0	0	1 (3)	0	0	0
Lung fibrosis	0	0	0	0	1 (6)	0	0	0	1 (3)	0	0	0
Tracheoesophageal fistula	0	0	0	0	1 (6)	0	0	1 (6)	1 (3)	0	0	1 (3)
Esophageal-mediastinal fistula	0	0	0	0	1 (6)	0	0	1 (6)	1 (3)	0	0	1 (3)

Note: Group A: Patients with R/SP-MLNMs who received treatment including surgery and/or chemotherapy, but not radiation therapy; Group B: Patients with R/SP-MLNMs who received treatment including surgery and/or chemotherapy, and radiation therapy

Percent was calculated based on the total enrolled in each group (group A = 16, group B = 17) or the total study (33).

## DISCUSSION

Locoregional recurrence of NSCLC after surgery occurs in approximately 20% of patients with stage I disease [29-30] and in up to 50% of patients with stage III disease [31-32]; the majority of failures are confined to the thorax. Currently, the NCCN guidelines recommended that patients with R/SP-MLNMs without prior RT receive chemo-RT because the same treatment approach would be used for inoperable MLNM patients. For patients who have already received RT, chemotherapy alone is recommended [11]. In the 1990s and 2000s, reports suggested that patients in patients who had disease involving the MLNs, the levels of disease are independent predictors of patient outcome; subsequently, clinicians have argued to treat these recurrences aggressively, though treatment approaches have been heterogenous [33-35]. This is the first study to evaluate the safety and efficacy of SRT (both SBRT and FSRT) for patients with R/SP-MLNMs from NSCLC.

In the present study, the median OS for patients with R/SP-MLNMs treated with SRT was 25.5 months (Figure 5A); this is superior to the OS 11-19 months in patients with R/SP-MLNM using conventional radiotherapy (3D-CRT or IMRT) [36-37], and 16-19 months in patients with unresectable IIIA and IIIB NSCLC receiving concurrent chemo-RT [38-40]. SRT achieves exciting LC rates for R/SP-MLNMs from NSCLC: 1-year and 3-year actuarial LC rates for all eligible patients were 100% and 85.5%, respectively, improved compared reported rates of conventional RT [36-37]. The reason for these discrepancies may be in part be attributed to the initial stage of patients (e.g. the Will Rogers phenomemon), the number of MLNMs involved, NSCLC histology, patient selection, and other treatments used.

In our series, patients with R/SP-MLNMs who had received prior RT had a shorter median OS compared to those without RT (15.3 months vs. 45 months, in Figure 5B), and this may be due to the more aggressive tumor biology, lower prescribed dose, lower dose per fraction, smaller PTV size, or lower BED_10_ used in Group B compared to those in Group A.

In addition, we found that the interval time between the surgery and SRT was important (Figure 5C). For patients who received SRT < 15.5 months after their surgery, the median OS was 42.0 months vs. 72.0 months for those treated at ≥ 15.5 months after surgery. In this retrospective series, a shorter time from surgery until SRT is likely a surrogate of more aggressive recurrent or second primary cancer. The time point of 15.5 months should not be interpreted as an “optimal time” window to deliver RT.

There was no significant difference in OS between patients with R-MLNMs vs. SP-MLNMs with respect to interval between surgery and SRT (Figure 5D). In addition, our results were consistent with that of some previous studies, which showed that the OS showed a slight trend towards superiority of SRT with chemo over SRT without chemo, although these differences were not statistically significant [34, 41-42]. We provide the details of chemotherapy in Table 1. Notably, the number of patients receiving chemotherapy was relatively small (24 patients before SRT; 14 patients after SRT), and these patients may have also received molecular targeted therapy (e.g. erlotinib for EGFR mutations). Chemotherapy regimens were tailored to individual patients: pemetrexed was preferred for adenocarcinoma, and a platinum in combination with gemcitabine was preferred for squamous cell carcinoma. Other agents used included paclitaxel, docetaxel, etoposide, and vinorelbine. The observed superiority of SRT with chemotherapy over SRT alone may be due to a number of factors, including (1) patient performance status and ability to tolerate chemotherapy (and the number of cycles of chemo); (2) histology and potential response to a particular agent; (3) molecular subtype and ability to use molecularly targeted therapy; and (4) synergy of chemotherapy and RT. Based on these considerations, we cannot recommend a particular systemic therapy regimen for all patients, and we encourage clinicians to use personalized approaches.

The treatment of patients with R/SP-MLNMs from NSCLC is a continuing challenge because the disease typically causes symptoms and is usually terminal. The common symptoms for our patients were cough, shortness of breath, hoarseness, and difficulty swallowing, which seriously affect quality of life. After SRT, symptoms improved after a median follow-up of 6 days (range, 3-18) for about half of patients, and there was continued alleviation throughout the follow-up period. Our findings concur with published data where palliative RT may improve pain, cough, hoarse, and dyspnea from recurrent NSCLC [35, 43-44].

In this study, no patient failed within the R/SP-MLNM PTV. Five patients (15%) had no progression after SRT (all in Group A), 17 (52%; 5 in Group A and 12 in Group B) who had progression, with a median of 16.9 months after SRT (range, 2.7-75.5 months). The most frequent sites of distant metastases were liver, lung, bone, brain, lymph nodes, and diffuse progression. It is interesting to note that the primary pattern of failure in this patient population would be local failure, had the patients received conventional RT [36, 45-46]. The reason for this discrepancy may be in part be attributed to the improved efficacy of SRT (compared to conventional RT), secondary to the higher prescribed dose, dose per fraction, and BED_10_. Based on the low incidence of loco-regional failure and high incidence of distant metastasis we observed, we would not recommend elective nodal irradiation in this patient population.

In the present study, SRT toxicities were mild (CTCAE Grade 1-2), with most patients experiencing pneumonitis, esophagitis, tracheitis, chest pain, agranulocytosis, and thrombocytopenia. These symptoms were transient and resolved with conservative management. More severe toxicities occurred more frequently in Group B; moreover, four patients with LN station 7 MLNMs receiving re-RT experienced tachycardia, lung fibrosis, tracheoesophageal fistula, and esophageal-mediastinal fistula. Importantly, 2 patients (6%) died from Grade 5 late toxicities. Both were treated to LN station 7: one patient died of tracheoesophageal fistula five weeks after completion of SRT; another patient died of tracheoesophageal fistula six weeks after completion of SRT.

A prospective multicenter dose-escalation trial, RTOG 0813, has provided some normal tissue constraints for consideration when treating tumors near sensitive central structures [25]. Additionally, clinical studies have demonstrated that RT dose, fractionation, dose-volume histogram constraints, as well as administration of systemic therapy are related to toxicity [47-49]. We caution clinicians to consider lymph node station (particularly zone 7) and use of previous RT before administration of SRT for R/SP-MLNMs.

This study had potential weaknesses. It is retrospective in nature, and it contains a relatively small number of patients who were treated over a long time period with heterogeneous fractionation regimens and systemic therapies. RTOG 0813 will help define the role of SBRT for patients with centrally-located lung tumors. We eagerly await the results of RTOG 0813, and we hope our clinical experience will complement its findings. In conclusion, to our knowledge, this is the first study to evaluate the safety and efficacy of SRT (both SBRT and FSRT) in the treatment of patients with R/SP-MLNMs from NSCLC. We recommend clinicians exercise extreme caution in using SRT for R/SP-MLNMs in patients who received prior RT (particularly to LN station 7). For patients without previous RT, SRT appears to be safe and efficacious treatment modality; prospective studies are warranted.

## PATIENTS AND METHODS

### Study design and eligible patients

We queried our prospectively-collected retrospective database of patients with R/SP-MLNMs originating from NSCLC. Patients were treated between October 1, 2006 and July 13, 2013. All patients were examined in a multidisciplinary setting by surgical (CLW), medical (KL), and radiation (MBM, ZYY, and PW) oncologists at the time of diagnosis, and their cases were re-presented in front of the tumor board on an as-needed basis (e.g. at time of recurrence).

The inclusion criteria were defined as follows: (i) any age; (ii) Karnofsky performance score (KPS) ≥ 70; (iii) R/SP-MLNMs from NSCLC with prior biopsy and histologic confirmation; and either computed tomography (CT) or positron emission tomography/computed tomography (PET-CT) images; (iv) life expectancy > 6 months; (vi) unamenable to resection (either because of anatomical tumor characteristics or patient comorbidities); and (vii) patient written informed consent for the treatment and database. We defined R-MLNMs as disease that was of the same histology as a previously-treated primary NSCLC. We defined SP-MLNMs as a different histology or having radiographic appearance inconsistent with progression of the original primary.

Exclusion criteria were as follows: contraindication to receiving RT; and uncontrolled comorbid condition (metabolic or psychiatric). The study protocol was in accordance with the ethical guidelines of the 1995 Declaration of Helsinki and was approved by the independent ethics committees at Tianjin Medical University Cancer Institute & Hospital, National Clinical Research Center for Cancer.

### Classification and delineation of R/SP-MLNMs

The MLN stations were classified according to the definition of Mountain and Dresler [19], and were delineated following the atlas from the University of Michigan [20]. MLNMs were divided into three groups: the upper mediastinal compartment, including stations 1, 2, 3, and 4; the middle mediastinal compartment, including stations 5 and 6; and the lower mediastinal compartment, including stations 7, 9, and 10.

### Treatment schedule

The methodology used for CyberKnife SRT and treatment planning has been described in detail in our previous reports [21-23]. Briefly, patients were immobilized using a vacuum bag before CT simulation. A set of planning three-dimensional (3D) and four-dimension (4D) CT images were obtained after injection of intravenous radiographic contrast material infusion to highlight the MLNMs. The gross target volume (GTV) was defined as the MLNM disease based on simulation, CT, and/or PET-CT. The planning target volume (PTV) was defined as the GTV with a margin of 0.3 cm in the x, y, and z-axis direction. The PTV was also amended to adjacent organs at risk (e.g. esophagus, heart). The Xsight spine tracking system was used for all targets, which carried out positional alignment based on bony spinal skeletal structures. Given the exploratory and retrospective nature of this project, and its span of seven years, the prescribed dose and fractionation were determined based on factors specific to each patient and cancer, including treatment interval since previous RT, patient performance status, target volume, and previous RT dose to the adjacent critical structures. Examples of each LN station dose distribution are shown in Figure 2.

Composite plans were generated of current and prior RT courses as previously described [18]. Dose volume histograms were analyzed, and if dose volume constraints were exceeded, clinical judgment was used to maximize the therapeutic ratio. Composite plans were generated as both an absolute summation of the two plans and with the treated doses converted to biologically effective doses (BEDs) and equivalent doses in 2 Gy fractions (EQD2s). BEDs were calculated based on the formula: nd[1 + d/(α/β)], where n is number of fractions, and d is dose/fraction (Gy); assuming α/β value of 10 for lung cancer or acute toxicities (i.e. BED_10_), and assuming α/β value of 3.0 for late toxicities (i.e. BED_3_). EQD2s were calculated based on the formula: nd((d+α/β)/(2+α/β)) [24].

MIM Software (v5.6, MIM Sofware Inc, Cleveland, OH) was used to convert dose to the EQD2, fuse scans, and combine the dose files. A sample of composite plan is shown in Figure 3. Normal tissue constraints for SRT planning were limited by constraints proposed by Kong et al., the Radiation Therapy Oncology Group (RTOG) 0236 and 0813 guidelines, and NRG BR-001 guidelines (provided in Supplementary materials 1) [20, 25-27]. In addition, if patients received chemotherapy, data was gathered about the agents used and the number of cycles.

### Follow-up

Patients were seen in clinic at 1 month after completion of treatment, then every 3 months for the first year; then, every 6 months until July 2013. Imaging, adverse events, and compliances of all patients were monitored for the follow-up period using our clinical databases.

### Endpoints

The primary end-point was overall survival (OS), defined as the time between the date of the SRT and the date of death or the date of the last follow-up for censored patients. The secondary end-points were: (1) MLNMs local control rate (LC; defined as no progression of treated disease on follow-up scans), either complete response (CR) or partial response (PR), defined using the RECIST 1.1 Response Evaluation Criteria in Solid Tumors [28], assessed at a minimum of 6 months of follow-up after SRT, in order to avoid uncertainty associated with early transient radiographic changes within the high-dose region; (2) the time to symptom alleviation (defined as the time between the date of the SRT completion and the date of symptom alleviation or the date of the last follow-up for censored patients); and (3) Common Terminology Criteria for Adverse Events (CTCAE v4.0) grade toxicity. All toxicities were assessed in a multidisciplinary setting. Patients were considered to have a local failure if there was evidence of increased size of enhancing tumor in the treated region. PET-CT scan was employed to assist with differentiating radiation related changes with local or regional recurrence.

### Statistical analysis

OS and LC curves were estimated using Kaplan-Meier analysis. Curves were compared by using the stratified log-rank test. A *p* value of 0.05 or less was considered statistically significant. Data were analyzed using the statistical software Intercooled Stata version 8.2 for Windows (Stata Corporation, College Station, Texas, USA).

## SUPPLEMENTARY MATERIAL


