# Supplementary material for: Clinical evaluation of stereotactic radiation therapy for recurrent or second primary mediastinal lymph node metastases originating from non-small cell lung cancer
Source: Oncotarget. 2015 Mar 30;6(17):15690–703. doi: 10.18632/oncotarget.3704 (PMC4558180; doi:10.18632/oncotarget.3704)
Supplement: Supplementary file 1 [file oncotarget-06-15690-s001.pdf]

Clinical evaluation of stereotactic radiation therapy for recurrent or second primary mediastinal lymph node metastases originating from non-small cell lung cancer

Supplementary Material

Supplementary material 1 The normal tissue constraints of the mediastinum for planning SRT

Organs/Tissue at risk	Dose limiting				
	RTOG 0236	RTOG 0813	NRG-BR001		Avoidance endpoint
Spinal cord	Volume: any point<br>Dose (cGy): 18 Gy (6 Gy/fx)	Volume: < 0.25 cc<br>< 0.5 cc<br>Volume max (Gy): 22.5 Gy (4.5 Gy/fx)<br>13.5 Gy (2.7 Gy/fx)<br>Max point dose (Gy): 30 Gy (6 Gy/fx)	3 fx:<br>Volume: < 0.03 cc<br>Volume dose (Gy): 22.5<br>Volume: < 1.2 cc<br>Volume dose (Gy): 13	5 fx:<br>Volume: <0.03 cc<br>Volume dose (Gy): 22.5<br>Volume: <0.35 cc<br>Volume dose (Gy): 22<br>Volume: <1.2 cc<br>Volume dose (Gy): 15.6	Myelitis
Esophagus	Volume: any point<br>Dose (cGy): 27 Gy (9 Gy/fx)	-	3 fx:<br>Volume: < 0.03 cc<br>Volume dose (Gy): 27<br>Volume: < 5 cc<br>Volume dose (Gy): 17.7	5 fx:<br>Volume: < 0.03 cc<br>Volume dose (Gy): 35<br>Volume: < 5 cc<br>Volume dose (Gy): 27.5	Stenosis/fistula
Ipsilateral brachial plexus	Volume: any point<br>Dose (cGy): 24 Gy (8 Gy/fx)	Volume: < 3 cc<br>Volume max (Gy): 30 Gy (6 Gy/fx)<br>Max point dose (Gy): 32 Gy (6.4 Gy/fx)	3 fx:<br>Volume: < 0.03 cc<br>Volume dose (Gy): 26<br>Volume: < 3 cc<br>Volume dose (Gy): 22	5 fx:<br>Volume: < 0.03 cc<br>Volume dose (Gy): 32<br>Volume: < 3 cc<br>Volume dose (Gy): 30	Neuropathy
Heart/Pericardium	Volume: any point<br>Dose (cGy): 30 Gy (10 Gy/fx)	-	3 fx:<br>Volume: < 0.03 cc<br>Volume dose (Gy): 30<br>Volume: < 15 cc<br>Volume dose (Gy): 24	5 fx:<br>Volume: < 0.03 cc<br>Volume dose (Gy): 38<br>Volume: < 15 cc<br>Volume dose (Gy): 32	Neuropathy

Organs/Tissue at risk	Dose limiting				
	RTOG 0236	RTOG 0813	NRG-BR001		Avoidance endpoint
Trachea and ipsilateral bronchus	Volume: any point<br>Dose (cGy): 30 Gy (10 Gy/fx)	-	3 fx:<br>Volume: < 0.03 cc<br>Volume dose (Gy): 30<br>Volume: < 5 cc<br>Volume dose (Gy): 25.8	5 fx:<br>Volume: < 0.03 cc<br>Volume dose (Gy): 40<br>Volume: < 5 cc<br>Volume dose (Gy): 32	Stenosis/fistula
Whole lung (right & left)	-	Volume: < 1000 cc<br>Volume max (Gy): 13.5 Gy (2.7 Gy/fx)<br>Max point dose (Gy): -	3 fx:<br><15% lung volume (Gy): 20<br><37% lung volume (Gy): 11	5 fx:<br><37% lung volume (Gy): 13.5<br><1500 cc (Gy): 12.5<br><1000 cc (Gy): 13.5	Pneumonitis
Skin	-	Volume: < 10 cc<br>Volume max (Gy): 30 Gy (6 Gy/fx)<br>Max point dose (Gy): 32 Gy (6.4 Gy/fx)	3 fx:<br>Volume: < 0.03 cc<br>Volume dose (Gy): 33<br>Volume: < 10 cc<br>Volume dose (Gy): 31	5 fx:<br>Volume: < 0.3 cc<br>Volume dose (Gy): 38.5<br>Volume: < 10 cc<br>Volume dose (Gy): 36.5	Ulceration
